# Reduction of intradiscal pressure by the use of polycarbonate-urethane rods as compared to titanium rods in posterior thoracolumbar spinal fixation

**DOI:** 10.1007/s10856-017-5953-0

**Published:** 2017-08-21

**Authors:** Eva Jacobs, Alex K. Roth, Jacobus J. Arts, Lodewijk W. van Rhijn, Paul C. Willems

**Affiliations:** Department of Orthopaedic Surgery, CAPHRI Care and Public Health Research Institute, Maastricht University Medical Centre, P. Debyelaan 25, 6229 HX Maastricht, The Netherlands

**Keywords:** Thoracolumbar hyperkyphosis, Intradiscal pressure, Polycarbonate-urethane, Axial stiffness, Spinal fixation

## Abstract

**Abstract:**

Loss of sagittal alignment and balance in adult spinal deformity can cause severe pain, disability and progressive neurological deficit. When conservative treatment has failed, spinal fusion using rigid instrumentation is currently the salvage treatment to stop further curve progression. However, fusion surgery is associated with high revision rates due to instrumentation failure and proximal junctional failure, especially if patients also suffer from osteoporosis. To address these drawbacks, a less rigid rod construct is proposed, which is hypothesized to provide a more gradual transition of force and load distribution over spinal segments in comparison to stiff titanium rods. In this study, the effect of variation in rod stiffness on the intradiscal pressure (IDP) of fixed spinal segments during flexion-compression loading was assessed. An ex vivo multisegment (porcine) flexion-compression spine test comparing rigid titanium rods with more flexible polycarbonate-urethane (PCU) rods was used. An increase in peak IDP was found for both the titanium and PCU instrumentation groups as compared to the uninstrumented controls. The peak IDP for the spines instrumented with the PCU rods was significantly lower in comparison to the titanium instrumentation group. These results demonstrated the differences in mechanical load transfer characteristics between PCU and titanium rod constructs when subjected to flexion-compression loading. The concept of stabilization with a less rigid rod may be an alternative to fusion with rigid instrumentation, with the aim of decreasing mechanical stress on the instrumented segments and the possible benefit of a decrease in the incidence of screw pullout.

**Graphical abstract:**

## Introduction

Vertebral body wedging may be the result of an anterior vertebral compression fracture or gradual deformation due to creep-like mechanisms [1]. Subsequent spinal deformity is often progressive and wedge accumulation over multiple thoracolumbar levels may lead to thoracolumbar hyperkyphosis. Factors such as (asymmetric) intervertebral disc degeneration, reduced spinal muscle strength, and weakening of the spinal ligament support also contribute to progressive thoracolumbar curvature [2, 3]. Inability to compensate for thoracolumbar hyperkyphosis leads to global spinal sagittal malalignment, which is the strongest driver of pain and disability [2, 4, 5].

Operative correction is not typically recommended as the first treatment option due to the high prevalence of osteoporosis and other medical comorbidities in the elderly patient population [2, 6, 7]. Instrumented spinal fusion is regarded more as an end-stage salvage procedure only reserved for patients suffering from intractable pain, severe disability, significant pulmonary function impairment or progressive neurological deficit not amenable to conservative treatment [3, 6–9]. This implies that patients considered for surgery are in an advanced stage of the degenerative process, with frequently compromised bone quality due to osteoporosis or osteopenia and/or severe global sagittal imbalance. As a result, surgery in these patients is associated with high rates of complications, such as proximal junctional failure (PJF), instrumentation breakout and recurrent spinal deformity [6, 8, 10]. In patients older than 55 years the risk of PJF is close to 40%, and as many as one-third of the patients undergo early revision within 1 year [6].

Bone quality is an important factor that influences stability of posterior spinal implants. Posterior instrumentation tends to fail due to limited screw-bone interface fixation strength in low-density bone, resulting in screw pullout, transitional fractures and loosening [11, 12]. Ohtori et al. [13] found that the incidence of screw pullout in the osteoporotic spine after 1- or 2-level posterolateral fusion for degenerative spondylolisthesis ranges from 15 to 25%. Conversely, the occurrence in young patients suffering from Scheuermann’s disease is relatively low and ranges from 0 to 3% [14]. Mechanical stiffness mismatch between rigid spinal instrumentation and osteoporotic bone contributes to the increased incidence of failure in the surgical treatment of hyperkyphosis in the elderly [2, 6, 14, 15].

The concept of ‘semi-rigid’ fixation has been introduced as an alternative to rigid metal constructs in order to minimize instrumentation related problems [14, 16–18]. A semi-rigid stabilization system would promote fusion via a balance of stability and intra- and inter-level load sharing [16, 19–21]. Polyetheretherketone (PEEK) rods have been proposed as such, as the Young’s modulus of PEEK is approximately 30-fold lower compared to Titanium (3.5 vs. 115 GPa, respectively) [22, 23]. Chou et al. [23] compared a PEEK rods construct to a titanium rods construct and found in PEEK rods a loading pattern more comparable to those of the intact spine based on the biomechanical changes of the disc height and IDP at both the instrumented and the adjacent levels. Moreover, rod stresses and bone stresses at the screw-bone interface measured by means of uniaxial strain gauges were reduced for the PEEK rod constructs, indicating a reduced risk of instrumentation failure [23].

Schmidt et al. [24] used finite element analysis to calculate the ideal axial and bending stiffness of a posterior stabilization device for different degrees of segmental stabilization. For semi-rigid fixation, defined as a reduction in range of motion up to 33%, the ideal axial stiffness was defined as approximately 45 N/mm, calculated as

$${\rm{c}}\left( a \right) = \frac{{{\rm{E}}A}}{l},\left(\begin{array}{c}\frac{{{\rm{N}}}}{mm}\end{array}\right)$$

where *l* is the length, A the area and E the Young’s modulus. The ideal bending stiffness was defined as approximately 30 N/mm and calculated as

$${\rm{c}}\left( b \right) = \frac{{3{\rm{E}}I}}{{{l^2}}},\left(\begin{array}{c}\frac{{{\rm{N}}}}{mm}\end{array}\right)$$

where *I* is the second moment of area [24]. Using PEEK as a material results in substantially higher stiffness values as required for semi-rigid fixation (axial stiffness 1386 N/mm, bending stiffness 131 N/mm) [23, 24]. This has been confirmed by Rohlmann et al. [25], who showed that only very low axial stiffness (<200 N/mm) of a posterior rod influences the segment kinematics. Therefore, we propose using a material with a much lower Young’s modulus for the early stage stabilization of (impending) thoracolumbar hyperkyphosis: polycarbonate-urethane (PCU). Polyurethane elastomers are typically composed of three reactive components: a diisocyanate, an oligomeric macromonomer and a chain extender [26], which can be combined in different ratios to produce polyurethane elastomers with vastly differing physicochemical and mechanical properties. The shore hardness of PCU is temperature dependent and reduces as temperature rises. The purpose of this study is to comparatively analyze the effect of spinal rod stiffness variation on intradiscal pressure (IDP) by means of PCU and titanium rod constructs subjected to dynamic flexion-compression loading. We hypothesize that a more flexible construct will result in reduced spinal load (intradiscal pressure). As reduced spinal load has been associated with reduced stresses at the screw-bone interface, the risk of instrumentation failure and PJF may thus potentially be reduced.

## Materials and methods

### Specimen preparation

**Fig. 1:**
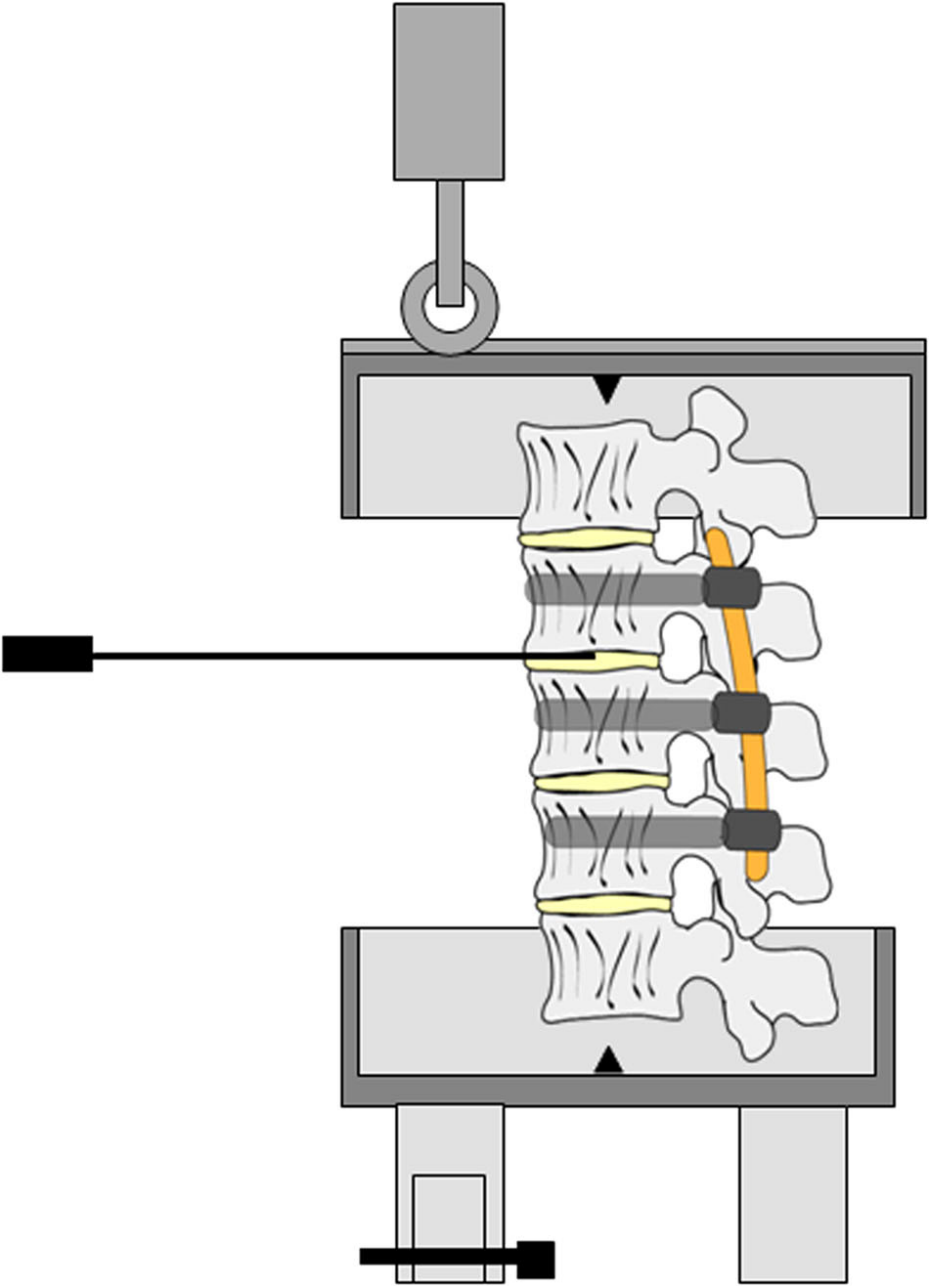
Schematic of the custom designed flexion-compression test set-up. The *lower* metal cup was firmly attached to the materials testing machine. The spinal segment was fixated by means of PMMA to the *lower* and *upper* metal cup. A roller was mounted on the vertical actuator of the materials testing machine and was forced to traverse the slot in the *upper* cup introducing a flexion movement of the spine. A counter balance mechanism was applied to return the spinal segment to its neutral position

Twenty porcine lumbar spine specimens were obtained from a local abattoir. At time of slaughter, all female animals weighed between 80 and 90 kilograms (10–14 months old). The spines were dissected and sectioned into multi-level segments from L1 to L5. All soft tissue was carefully removed leaving the ligaments, intervertebral discs and facet joint capsules intact. Radiographs of the porcine spines were made to exclude any anatomic abnormalities. The porcine specimens used in the current study showed normal spine morphology without radiologic evidence of any spinal pathology. After preparation, the specimens were wrapped in saline soaked gauze, sealed in plastic bags and stored at −18 °C. The segments were thawed in a 4 °C environment to acclimate to room temperature approximately 24 h prior to testing. The porcine cadaveric specimens underwent only one freeze-thaw cycle, which according to the literature has no negative effect on the biomechanical properties of the spine [27–29]. The upper and lower half of respectively vertebrae L1 and L5 were both fixed in cups fitting the test setup (Fig. 1), using polymethylmethacrylate (PMMA, Technovit 3040, Heraeus, Hanau, Germany). Screws were driven into the endplates of the cranial (L1) and caudal (L5) vertebrae to improve fixation between the PMMA and the vertebrae.

### Specimen instrumentation

Four different groups were tested: (1) uninstrumented spines (*n* = 5), pedicle screw instrumentation with (2) titanium rod fixation (*n* = 5), (3) PCU (Bionate®, DSM Biomedical, Geleen, the Netherlands) shore hardness 75D rod fixation (*n* = 5), and (4) PCU shore Hardness 65D rod fixation (*n* = 5). The Young’s modulus of the different rods was 115,000 N/mm^2^ for the titanium rods, 188 N/mm^2^ for the 75D PCU rods and 120 N/mm^2^ for the 65 PCU rods at 37 °C. The PCU rods were rehydrated in a saline bath at 37 °C for at least 48 h prior to implantation. Posterior instrumentation spanning three motion segments was applied to fifteen fresh porcine spinal segments (L2, L3 and L4) by use of standard surgical technique. Six mono-axial titanium alloy pedicle screws (30-mm length, 5.5-mm diameter, Medtronic Inc., Minneapolis, MN) were inserted transpedicularly without wall breach under fluoroscopic guidance. Plain anteroposterior and lateral radiographs were taken to confirm correct placement of the pedicle screws and to exclude bone abnormalities. Screw-rod instrumentation was applied to the L2 to L4 segment consisting of the aforementioned six pedicle screws and either the PCU or titanium alloy rods, all 120 mm in length and 5.5 mm in diameter. The use of PCU rods with these dimensions allowed for a fundamental comparison. Table 1 shows an overview of the material properties for the different rods used, including the length, diameter, Young’s modulus, axial stiffness and bending stiffness.

**Table 1:** Overview of the material properties of the rods (mean ± standard deviation)

	Length (cm)	Diameter (mm)	Young’s modulus (N/mm^2^)	Axial stiffness (N/mm)	Bending stiffness (N/mm)
Titanium	12	5.5	115,000	22,768	1076
PCU 75D	12	5.5	188 ± 30	37	2
PCU 65D	12	5.5	120 ± 20	24	1

Overview of the material properties of the rods (mean ± standard deviation)

### Mechanical testing

A combined flexion-compression load was applied to the spinal segments by means of an eccentric force [30]. The tests were conducted in a custom designed test setup placed in a saline bath at 37 °C in order to mimic body temperature and to account for the temperature dependent material properties of the PCU rod (Fig. 1). The lower metal cup was firmly attached to the materials testing machine (Zwick/Roell, ZMART PRO) after fixation of the spinal segment in PMMA. A roller was mounted on the vertical actuator of the materials testing machine containing a load cell. When the load was applied, the roller was forced to traverse the slot in the upper cup in the anterior-posterior direction, thereby preventing out-of-plane bending. A counter balance mechanism was applied in order to return the spinal segment to its neutral position. The vertical actuator applied a 115 N load, 65-mm anterior to the midpoint of the L1 vertebral body, without preload, creating flexion combined with axial compression of the specimen [27]. The load was applied with a vertical actuator speed of 400-mm/min for a total of 3.000 cycles.

### Intradiscal pressure measurements

A 1.3-mm diameter pressure transducer (8CT/4F/SS/HP, Gaeltec Ltd, linearity error <1 ± 1%) was used to measure IDP. The annulus was punctured using an 18 gauge needle, and the transducer was inserted into the L2-L3 disc before testing. Continuous measurement was performed.

During loading of the spinal segment, IDP was measured and the average results were calculated after 0, 100, 250, 500, 1.000, 1.500, 2.000, 2.500, and 3.000 loading cycles, respectively.

### Statistical analysis

Statistical analysis was performed on IDP data using SPSS version 20 for Windows (SPSS, Inc., Chicago, IL). Mean data obtained during sagittal plane testing were compared between intact and different instrumented testing using a Mann-Whitney U test (significance level at *P* < 0.05). Since this study is a concept study a post-hoc power analysis was performed.

## Results

**Fig. 2:**
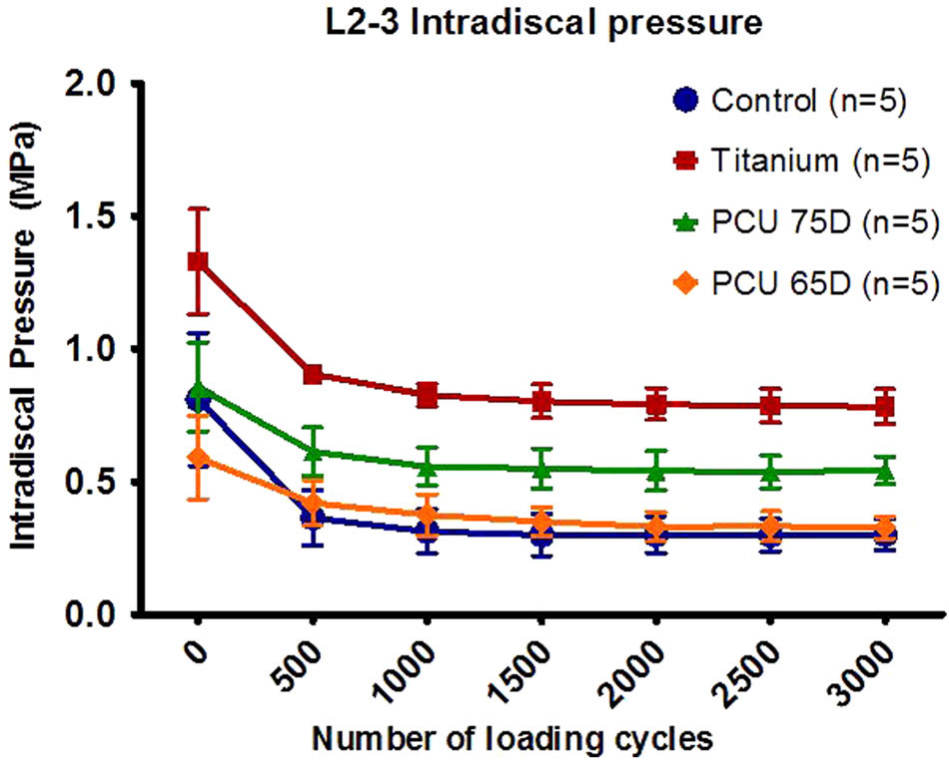
Mean L2-L3 intradiscal pressures (IDP) within instrumented segments L2-L3. The error bars represent the mean ± standard deviation of 5 independent measurements for all three instrumented groups and the uninstrumented control group

Stabilization of the viscoelastic response of the spines occurred after approximately 1.500 cycles of loading for all testing conditions (Fig. 2). Peak IDP at the L2-L3 level for all groups is shown in Table 2 and Fig. 2.

**Table 2:** Summary of the intradiscal pressure results in MPa (mean ± standard deviation) for the uninstrumented lumbar spines and the groups with titanium or PCU 65D and 75D instrumentation, respectively

Group	Number of loading cycles						
	0	500	1000	1500	2000	2500	3000
Control (*n* = 5)	0.81 ± 0.25	0.36 ± 0.10	0.31 ± 0.08	0.30 ± 0.08	0.30 ± 0.07	0.30 ± 0.06	0.30 ± 0.06
Titanium (*n* = 5)	1.33 ± 0.20^*^	0.90 ± 0.02^*^	0.83 ± 0.04^*^	0.80 ± 0.06^*^	0.79 ± 0.06^*^	0.79 ± 0.06^*^	0.78 ± 0.06^*^
PCU 75D (*n* = 5)	0.86 ± 0.17	0.61 ± 0.09^**^	0.56 ± 0.07^**^	0.55 ± 0.08^**^	0.54 ± 0.07^**^	0.54 ± 0.06^**^	0.54 ± 0.05^**^
PCU 65D (*n* = 5)	0.59 ± 0.16	0.42 ± 0.09	0.38 ± 0.08	0.35 ± 0.05	0.33 ± 0.06	0.33 ± 0.06	0.33 ± 0.04

The * represents the significant difference between the titanium instrumented spines and the uninstrumented and PCU instrumented spines (*P* = 0.009). The ** represents the significant difference between the 75D PCU instrumented spines and the spines instrumented with a 65D PCU rod or the uninstrumented spines (*P* < 0.05)

The initial peak IDP, at 0 cycles, increased in the presence of instrumentation. The greatest significant increase in initial IDP as compared to the uninstrumented spines, was found in the titanium instrumented group (1.33 vs. 0.81 MPa, *P* = 0.009). This was not the case for the 75D and 65D PCU instrumented spines (0.86 and 0.59 MPa respectively vs. 0.81 MPa, *P* > 0.05).

The uninstrumented and PCU instrumented spines consistently produced significantly lower IDPs throughout the test compared to the titanium instrumented spines (*P* < 0.05). After 500 cycles the peak IDP also increased significantly for the 75D PCU instrumented spines in comparison to the uninstrumented spines (*P* < 0.05).

After 3.000 cycles the IDP for spines instrumented with a titanium rod was significantly higher than that of all other groups (*P* = 0.009). For the spines instrumented with a 75D PCU rod the IDP after 3.000 cycles was significantly higher in comparison to the spines instrumented with a 65D PCU rod or the uninstrumented spines (0.54 vs. 0.33 and 0.30 respectively, *P* = 0.009). There was no significant difference in IDP between the 65D PCU rod and the uninstrumented spines at 3000 loading cycles (*P* = 0.347).

The results of the post-hoc power analysis indicated that the sample size of five spines per group is sufficient to find a significant difference between the different groups. Therefore the chosen sample size was determined to be adequate considering the objective of the concept study and the consistency in the biomechanical output.

## Discussion

Spinal fusion using long segment rigid instrumentation is currently the only surgical treatment to correct or stop curve progression in patients suffering from severe thoracolumbar hyperkyphosis [2, 3, 7]. Instrumentation failure or transitional fractures often occur as a result of diminished bone quality or sagittal imbalance of the patient, which leads to high rates of revision surgery [6, 8, 14]. Some of these complications can be attributed to high device stiffness. In this study, we assessed a conceptually new rod construct comprised of PCU with lower axial stiffness. The purpose was to obtain more fundamental insight into the effect of spinal rod stiffness variation on mechanical load transfer in the spine using PCU and titanium rods during cyclic flexion- compression loading.

The PCU rods construct demonstrated more comparable intradiscal pressure (IDP) outcomes to those of the intact spine when compared with the titanium rods construct, indicating a more physiological load distribution. The IDP, a direct measurement of mechanical load transfer through the anterior spinal column, is dependent on the range of motion of the spine [40]. The use of posterior instrumentation causes the center of rotation to move posteriorly beyond the posterior vertebral body line and superiorly into the disc space [21]. This posterior shift of the center of rotation causes axial compression and a higher loading of the intervertebral disc during flexion (i.e. higher IDP) [20, 31, 32]. Schilling et al. [20] studied the influence of design parameters of different pedicle screw based motion preservation systems on load transfer within the intervertebral disc and concluded that implant axial stiffness was the most important parameter. In their in vitro study low axial stiffness resulted in a lower amount of stabilization in the sagittal plane in comparison to stabilization provided by instrumentation with high axial stiffness [20]. This has been confirmed by Jahng et al. [21], whose finite element model showed that the shift in center of rotation differs according to the design and properties of the materials used for pedicle-based dynamic stabilization systems. The more rigid the pedicle-based dynamic stabilization device, the more the instantaneous center of rotation is shifted posteriorly and thus the nucleus pulposus is subjected to higher compressive loads. The interdependency between the axial stiffness of a posterior device on the amount of load transfer in the intervertebral disc is also confirmed by the significant difference in peak IDP between the 75D and 65D PCU instrumented groups in the current study. The axial stiffness of the 5.5-mm diameter rods used in the current study was calculated to be 22,768 N/mm for titanium rods, 37 N/mm for 75D PCU rods and 24 N/mm for 65D PCU rods at 37 °C according to equation 1. The axial stiffnesses of the PCU rods are within the range of a semi-rigid implant defined by Schmidt et al. [26]. It is therefore expected that both PCU rods will reduce the spinal flexibility (range of motion) up to only 33% compared to the non-fixated situation, whereas the rigid titanium rod will provide a maximum reduction in spinal flexibility of 66% compared to the non-fixated situation. These results indicate that when using spinal implants with low stiffness there is still some degree of stabilization of the spine combined with a loading pattern more comparable with the intact, uninstrumented spine.

The bending stiffness of the rods used in the current study, as calculated according to equation 2, are approximately 1076 N/mm for titanium rods, 2 N/mm for 75D PCU rods and 1 N/mm for 65D PCU rods at 37 °C. The bending stiffness of the PCU rods, in contrast to the axial stiffness, are outside the range of a semi-rigid rod as defined by Schmidt et al., being approximately 15 to 30 times lower [24]. In flexion and extension the effect of bending stiffness on the segmental stabilization is negligible, however, it is of high importance in lateral bending and axial rotation. According to the beam theory derived by Euler and Bernoulli, low bending stiffness increases the risk of buckling when an axial force is applied, since the critical force needed for buckling is proportional to the moment of inertia and the modulus of elasticity. By altering the geometry and/or elasticity of the rod the risk of buckling might be decreased. The PCU rods used in this study served the sole purpose of providing more fundamental insight on the effect of rod stiffness variation on the IDP. It still remains unclear which exact axial and bending stiffness and rod geometry will prove to be the most successful for less rigid stabilization with the optimal balance between attained correction and a minimal raise in load transfer through the instrumented spinal segments in patients suffering from thoracolumbar hyperkyphosis [20, 24].

Chou et al. [23], compared the effect of PEEK vs. titanium rod constructs on the disc height and IDP in the instrumented and adjacent levels after fatigue loading. A reduction of load transfer at the instrumented level subsequently resulted in an increased loading through the adjacent levels in titanium instrumented spines. PEEK rods were found to have a better capacity to reduce the non-physiological loadings at the cranial and caudal levels leading to a lower incidence of adjacent level failure [23]. The PCU rods used in the current study showed a comparable biomechanical pattern to the PEEK rods used by Chou et al. [23], and sustained a loading pattern more consistent with the uninstrumented spinal segment test group. This is of high clinical importance since pressures within normal physiological range are critical in providing a suitable environment to prevent instrument failure or proximal junctional failure (PJF) [33]. Based on the results of the current study it is expected that less rigid rods will allow better load sharing among spinal components leading to less junctional segment failure.

Mechanical stiffness mismatch between rigid spinal instrumentation and low mineral density bone has been suggested to contribute to high failure rates [8, 10, 12]. One of the goals of a more flexible rod is minimizing load transfer and subsequent stresses at the bone-screw interface. A similar pattern as found by Chou et al. [23], who confirmed that the stress on PEEK rods and the bone stresses near the bone-screw interface were significantly lower compared to titanium, is expected to apply for PCU rods [34]. However, Galbusera et al. [35] described a tendency to high screw loosening rates after stabilization with a less rigid rod in their literature review based on a study of Schatzker et al. [34]. This was explained by the absence of bony fusion leading to micromotion and formation of fibrous tissue around the screws [35, 36]. In our opinion, in order to circumvent this problem, screw augmentation with bone cement or fixation with laminar wires instead of pedicle screws could be used in conjunction with the less rigid PCU rods in the osteoporotic patient population [35, 37].

The present study has some limitations. First, only flexion-compression loading was tested in this study. This is due to the fact that hyperkyphosis is a single plane deformity in which the flexion movement of the spine is affected the most. In future research, a more advanced spinal loading simulator should be used which is capable of simulating flexion/extension moments, lateral bending moments and axial rotation [27]. Second, the global range of motion was not measured, which made analysis of standard moment-rotation curves not possible [38]. The IDP provides a direct measurement of mechanical load transfer in the spine. Therefore, measurement of the full range of motion would be better to quantify the acute stabilization effects of posterior pedicle screw instrumentation in comparison to the uninstrumented spine. Third, the effect of rod stiffness variation on mechanical load transfer in the adjacent discs and stresses on the bone-screw interface were not assessed. Fourth, the inherent motion properties of the specimen were not measured before testing the stabilizing capacities of the implant systems. For future research this is necessary in order to assure that mechanically defective specimens are excluded and to provide an individual basis for normalization [27]. Fifth, the effect of stress relaxation and creep of the polymer components was not assessed. To this end, at least 180,000 cycles, representative for 6 weeks in vivo human movement, should be carried out [39]. In our study long-term cycling was impossible to test due to the high decomposition rate of the cadaveric specimens at 37 °C. However, Bionate® II PCU is currently being used in a wide range of applications, including neurostimulation, vascular, artificial heart, and in various orthopedic applications. It is one of the most extensively tested families of biomedical polymers and is backed by a comprehensive FDA master file. Bionate® II PCU is proven to be biostable and biocompatible. At last, quadrupeds have often been used for spinal research [40]. The porcine spine has, in specific situations, proven to be the most representative biomechanical model for the human spine [41, 42]. When spinal instrumentation is tested in the porcine, some general differences need to be kept in mind. The axial compression stress in quadrupeds is higher, which leads to higher bone mineral densities in the vertebrae. Due to the higher bone mineral density, the porcine bone is harder compared to the human osteoporotic bone. This results in a higher fixation strength for pedicle screws inserted into porcine vertebrae [40]. The porcine spine shows a less pronounced thoracic kyphosis and lumbar lordosis, compared to the human spine [42]. Moreover, the lumbar porcine spine is slightly kyphotic. The range of motion, particularly in flexion/extension, is over all levels much smaller compared to the human range of motion [43]. This is mainly due to the different orientation of the facet joints. The facet joint surfaces of the porcine spine are oriented at an angle of less than 30 degrees relative to the frontal plane compared to an angle of more than 60 degrees in the human spine [41, 42, 44]. Thoracolumbar hyperkyphosis mainly affects the thoracolumbar part of the human spine. However, the porcine thoracic region is not comparable to the human thoracic region due to differences in anatomical dimensions, facet joint orientation and length of spinous process. In the porcine, the anterioposterior vertebral depth and vertebral body width are smaller compared to the respective human dimensions. Dath et al. described the measurements for the width and depth of the lumbar porcine vertebrae being approximately 35 and 25 mm, respectively [45]. These values are comparable with the human thoracic vertebrae (width T7-T10 29–35 mm, depth 28–32 mm) as described by Bozkus et al. [46]. Therefore, in absolute values, the size of the vertebrae are more comparable in the lumbar region of the porcine [42, 47]. The length of the spinous process is in the thoracic region of the porcine spine two to three times longer than the spinous process in the human spine [42]. In order to answer our specific research question, we considered the lumbar porcine spine suitable to predict the behaviour of the human spine. Most importantly, in the current study the effect of variation in rod stiffness on the intradiscal pressure of instrumented segments was assessed in a uniform, homogeneous spine model providing comparable biomechanical properties and geometry for each spine used.

The results of the current study suggest that dynamic fixation might lead to a decreased load on the bone screw interface, which may prevent instrumentation- and adjacent segment failure in the surgical treatment of degenerative spinal deformity. Further work to objectively compare pedicle screw pullout in rigid constructs and PCU constructs is currently ongoing in our laboratories.

## Conclusion

In this concept study, the effect of spinal rod stiffness variation of a posterior stabilization device on the intradiscal pressure during cyclic flexion-compression loading conditions was assessed. Low implant stiffness was found to generate lower IDP, representing a more physiological loading pattern in comparison to high implant stiffness. Since a reduced spinal load has been associated with decreased stress at the screw-bone interface, the risk of instrumentation failure and PJF may potentially be reduced when using a stabilization device with lower implant stiffness. However, the optimal rod stiffness, achieving a predefined stabilization of the spine, remains unknown and requires further investigation.
